# Oral Mucosal Melanoma and Sinonasal Amelanotic Melanoma: A Summary of Two Unusual Cases

**DOI:** 10.30699/ijp.2024.2014328.3199

**Published:** 2024-10-15

**Authors:** Krishnendu Mondal, Rupali Mandal

**Affiliations:** *Department of Laboratory Medicine, Woodland Hospital, Shillong, Meghalaya, India*

Dear Editor, 

Mucosal melanoma of the head and neck (MMHN) region accounts for ~1.3% of all melanomas affecting the body (1). The conjunctiva is most frequently involved, followed by the upper aerodigestive tract. The oral and nasal cavities share almost 48% and 44% of the melanomas occurring in the upper aerodigestive tract. Paranasal sinuses harbor the bulk of the remainder of cases. Rarely are the pharynx and larynx involved (2). Nearly 80-90% of oral mucosal melanomas (OMM) arise from the keratinizing mucosa of the hard palate and maxillary gingiva. Buccal mucosa, mandibular gingiva, and the floor of the mouth are rather unusual sites (3). The amelanotic version of melanoma constitutes ~13% of all MMHN, which is more than its cutaneous incidence of 1.8-8.1% (4). 

**Case 1**

The 42-year-old lady presented with an irregular blackish mucosal patch involving the inner surface of the upper lip (Figure 1A). Any pain or tenderness was absent. Only mere discomfort to the part led to its self-localization. Palpably, the mucosa appeared slightly thickened. Clinically, hemangioma and malignant melanoma surfaced as possible differentials. On histopathology: The thinned-out mucosa overlying diffuse sweeps of heavily pigmented tumor cells (Fig. 1B). These epithelioid tumor cells contained abundant cytoplasm, flocked with dense pigment granules that eventually obliterated their cellular details. Nuclei appeared vesicular with prominent nucleoli. Histomorphologically, the diagnosis of malignant melanoma was evident. Simultaneous positron emission tomography (PET) negated any further dissemination of the melanoma. Postoperatively, after a regimen of radiotherapy and chemotherapy the patient was followed up at 6 months interval without any relapse or recurrence. 

**Case 2**

A 56-year-old man attended the otorhinolaryngology outpatient clinic with a rapidly enlarging left paranasal mass on his face. Computed tomography (CT) delineated a homogeneous solid tumor arising from the maxillary sinus, encroaching and compressing onto the nasal cavity (Fig. 1C). With a primary suspicion of sinonasal carcinoma or lymphoma, a punch biopsy was taken from the mass. Histologically, undifferentiated tumor cells appeared in diffuse sweeps undermining the ulcerated mucosa (Figure 1D, 1E). The polygonal tumor cells bore grossly irregular large nuclei, marked nuclear pleomorphism, frequent intranuclear cytoplasmic pseudoinclusions, vesicular chromatin, prominent nucleoli, and abundant pale eosinophilic cytoplasm (Fig. 1F). From such a dubious histomorphology sinonasal undifferentiated carcinoma, poorly differentiated squamous cell carcinoma (SCC), large B-cell lymphoma and amelanotic form of malignant melanoma posed as closest differentials. To their discrimination, a battery of immunohistochemical (IHC) markers were exercised. Cytokeratin (CK) 5/6, CD 45, and S100 were applied in the primary panel. The tumor cells expressed strong nucleocytoplasmic reactivity with S100, indicative of malignant melanoma (Figure 1G). While the other two reagents stained negatively (Figure 1I, 1J). HMB 45 was applied for confirmation. Tumor cells expressed strong cytoplasmic granular positivity, affirming it as amelanotic melanoma (Figure 1H). PET scan detected widespread dissemination to cervical lymph nodes, brain and lungs. The patient was immediately subjected to combination therapy. Despite this, he died of the disease after 4 months. 

**Fig. 1:**
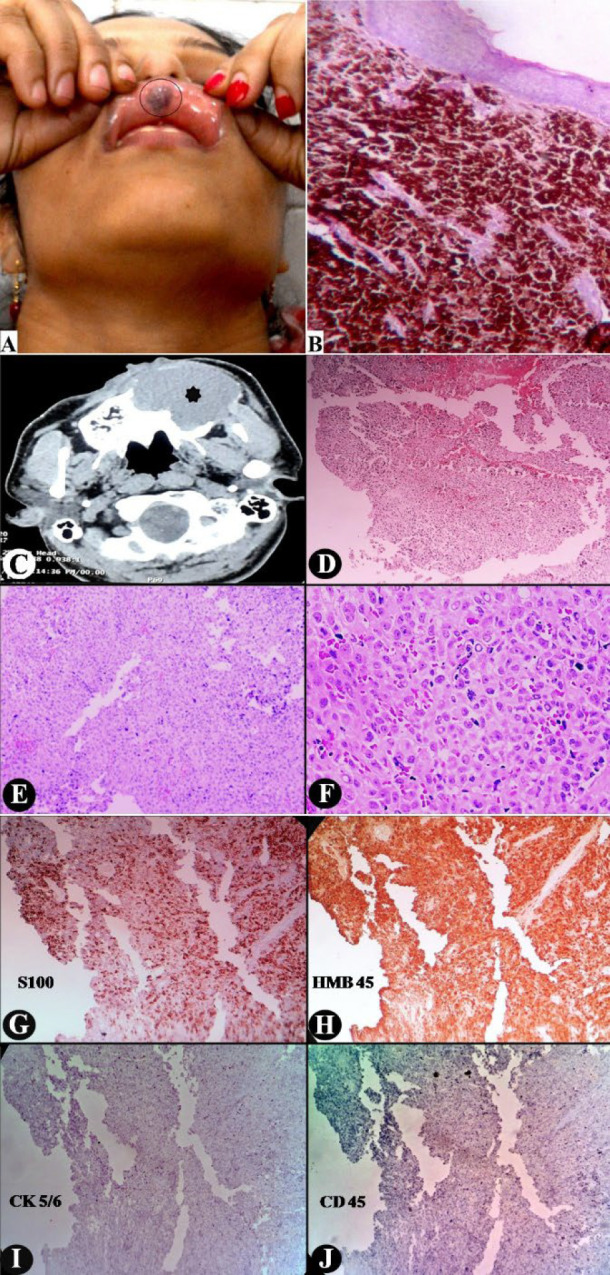
***Case 1:*** Macroscopically, melanotic mucosal patch (circle) on inner aspect of the upper lip (A). Microscopically, diffuse sheets of melanoma cells underlying the thinned-out mucosal stratified squamous epithelium (B) [Haematoxylin-Eosin stain, ×100].

***Case 2:*** Radiologically, a solid mass (asterisk) arising from left maxillary sinus (C). Microscopically, undifferentiated polygonal tumor cells in solid sheets [(D: Haematoxylin-Eosin stain, ×40) and (E: Haematoxylin-Eosin stain, ×100)]. On magnification, pleomorphic large tumor cells bear intranuclear inclusions, vesicular chromatin and prominent nucleoli (F) [Haematoxylin-Eosin stain, ×400]. Immunohistochemically, the tumor cells stain positively with S100 (G) and HMB 45 (H), but remain non-reactive with cytokeratin 5/6 (I) and CD 45 (J) [Immunohistochemistry, ×100].

Melanocytes of neural crest origin undergo malignant transformation into melanoma. Sinonasal location is a rare site for MMHNs, representing about 4% of all sinonasal tumors. At the oral cavity, it accounts for around 0.5% of all malignancies there (2, 4). Mucosal melanoma is unique from its cutaneous counterparts in risk association, pathogenesis, cytogenetics, and adverse tumor progression. It carries a poorer prognosis, with 5-year survival seen in only 30% of patients. MMHN usually affects elders in their sixth-seventh decades of life (4, 5). However, patients usually suffer from OMM during their 30-60 years of age (2). Both the patients described in this report coincided with similar age distribution. The lady with a localized buccal lesion had a better outcome. On the contrary, the patient with sinonasal melanoma simultaneously suffered from multiorgan spread and inadvertently died from the disease.

Because of its inert location, sinonasal melanomas often develop and progress insidiously with minimal symptomatology. Symptoms, if any, appear unilaterally with stuffy nose, runny nose, epistaxis, hyposmia, facial pain, proptosis, epiphora, frontal headache or diplopia. Nodal or distant spread is common by the time of presentation (6). The currently reported patient with sinonasal melanoma complained about the facial deformity caused by mass effect. Any other symptoms he ignored. Therefore, he presented late with the disease. By the time he developed widespread nodal and distant metastases, which eventually cost his life within 4 months.

OMM mainly arises *de novo*. Sometimes it may supervene on long-standing benign pigmentary lesion. Generally, it remains asymptomatic. Ulceration, tumoriform growth, bleeding and pain manifest as late symptoms (7). Clinically it appears as flattened pigmented patch of variable thickness. Overall deceptive presentation makes OMM masquerade with rare oral melanotic macule, nevus, smoker’s melanosis, amalgam tattoo, or Kaposi’s sarcoma. Hence, histopathology is essential for ultimate discrimination (2). Similarly, the discussed patient of oral melanoma experienced an asymptomatic course until a negligible mucosal discomfort led her to self-explore and eventual discovery of the lesion. Conventional histopathology confirmed the diagnosis of malignant melanoma for her. 

The pigmented form of malignant melanoma never poses a diagnostic difficulty, like the case of OMM discussed in this present report. However, diagnosing amelanotic melanoma in the sinonasal cavity is difficult. Morphologically, the melanoma cells may appear epithelioid or pleomorphic, simulating sinonasal undifferentiated carcinoma, poorly differentiated squamous cell carcinoma, or large B-cell lymphoma; they may become spindly mimicking undifferentiated sarcoma or can presume undifferentiated small cell morphology reminiscing olfactory neuroblastoma, small cell carcinoma or non-Hodgkin lymphoma (1, 6). A similar dilemma was again confronted with the present case of sinonasal amelanotic melanoma. Microscopically, the undifferentiated polyhedral tumor cells appeared in diffuse sweeps. Its overall histomorphology reminisced sinonasal undifferentiated carcinoma, poorly differentiated SCC, large B-cell lymphoma, and amelanotic melanoma as latent possibilities. CK 5/6 and CD 45 were strongly negative in the tumor cells, thus respectively excluding undifferentiated carcinoma and SCC, and also the lymphoma. Whereas S 100, followed by HMB 45, stained strongly in the tumor cells, confirming the tumor as melanoma. 

Although the prognostication of both cutaneous and mucosal melanomas is similar, MMHNs present at more advanced stages. Its TNM staging commences from T3 onwards (3, 8). The widely accepted Clark’s staging for cutaneous melanoma is irrelevant to the mucosa because of the disparate mucosal histology. Also, the MMHNs are relatively chemoresistant. Hence, a consensus regarding its therapeutic approach is yet elusive (9). For localized MMHNs, a wide excision with sufficient negative margins is the treatment of choice. The inclusion of neck dissection remains at the surgeon’s behest. Postoperatively, radiotherapy, chemotherapy, and immunotherapy are applied as adjuvant. Radiotherapy is useful for containing melanoma locally. Whereas chemotherapy and immunotherapy suppress its distant spread. For inoperable advanced MMHNs, such combination therapy brings about the best overall outcome (2, 3, 9). An indifferent therapeutic protocol was followed in both the present cases. The lady suffered from a localized OMM of the upper lip without any dissemination. She underwent wide excision combined with chemo-irradiation and continued for 6 months of recurrence-free survival. The other patient was promptly treated with combination therapy for the widely disseminated sinonasal melanoma. But he was overwhelmed by the ultimate fatality of the disease.

## References

[1] Williams MD, Speight P, Wenig BM, In: El-Naggar AK, Chan JKC, Grandis JR, Takata T, Slootweg PJ (2017). Mucosal melanoma. WHO Classification of Head and Neck Tumours.

[2] Mohan M, Sukhadia VY, Pai D, Bhat S (2013). Oral malignant melanoma: Systematic review of literature and report of two cases. Oral Surg Oral Med Oral Pathol Oral Radiol.

[3] Aloua R, Kaouani A, Kerdoud O, Salissou I, Slimani F (2021). Melanoma of the oral cavity: A silent killer. Ann Med Surg (Lond).

[4] Pontes FS, de Souza LL, de Abreu MC, Fernandes LA, Rodrigues AL, do Nascimento DM (2020). Sinonasal melanoma: A systematic review of the prognostic factors. Int J Oral Maxillofac Surg.

[5] Breik O, Sim F, Wong T, Nastri A, Iseli TA, Wiesenfeld D (2016). Survival outcomes of mucosal melanoma in the head and neck: Case series and review of current treatment guidelines. J Oral Maxillofac Surg.

[6] Alves ISS, Berriel LGS, Alves RT, Pinto MB, Oliveira CFP, Cazzotto AC (2017). Sinonasal melanoma: A case report and literature review. Case Rep Oncol Med.

[7] Shen ZY, Liu W, Bao ZX, Zhou ZT, Wang LZ (2011). Oral melanotic macule and primary oral malignant melanoma: Epidemiology, location involved, and clinical implications. Oral Surg Oral Med Oral Pathol Oral Radiol Endod.

[8] Lee G, Baek CH, Choi NY, Chung MK (2017). The prognostic role of the surgical approach and adjuvant therapy in operable mucosal melanoma of the head and neck. Clin Exp Otorhinolaryngol.

[9] Bhullar RP, Bhullar A, Vanaki SS, Puranik RS, Sudhakara M, Kamat MS (2012). Primary melanoma of oral mucosa: A case report and review of literature. Dent Res J (Isfahan).

